# 3D Printing—Encompassing the Facets of Dentistry

**DOI:** 10.3389/fbioe.2018.00172

**Published:** 2018-11-22

**Authors:** Gunpreet Oberoi, Sophie Nitsch, Michael Edelmayer, Klara Janjić, Anna Sonja Müller, Hermann Agis

**Affiliations:** ^1^Department of Conservative Dentistry and Periodontology, University Clinic of Dentistry, Medical University of Vienna, Vienna, Austria; ^2^Austrian Cluster for Tissue Regeneration, Vienna, Austria; ^3^Center for Medical Physics and Biomedical Engineering, Medical University Vienna, Vienna, Austria; ^4^Department of Health Sciences, FH Wien, University of Applied Sciences, Vienna, Austria; ^5^Department of Oral Surgery, University Clinic of Dentistry, Medical University of Vienna, Vienna, Austria

**Keywords:** 3D printing, additive manufacturing, bioprinting, dentistry, education, tissue engineering

## Abstract

This narrative review presents an overview on the currently available 3D printing technologies and their utilization in experimental, clinical and educational facets, from the perspective of different specialties of dentistry, including oral and maxillofacial surgery, orthodontics, endodontics, prosthodontics, and periodontics. It covers research and innovation, treatment modalities, education and training, employing the rapidly developing 3D printing process. Research-oriented advancement in 3D printing in dentistry is witnessed by the rising number of publications on this topic. Visualization of treatment outcomes makes it a promising clinical tool. Educational programs utilizing 3D-printed models stimulate training of dental skills in students and trainees. 3D printing has enormous potential to ameliorate oral health care in research, clinical treatment, and education in dentistry.

## Introduction

**Figure 1:**
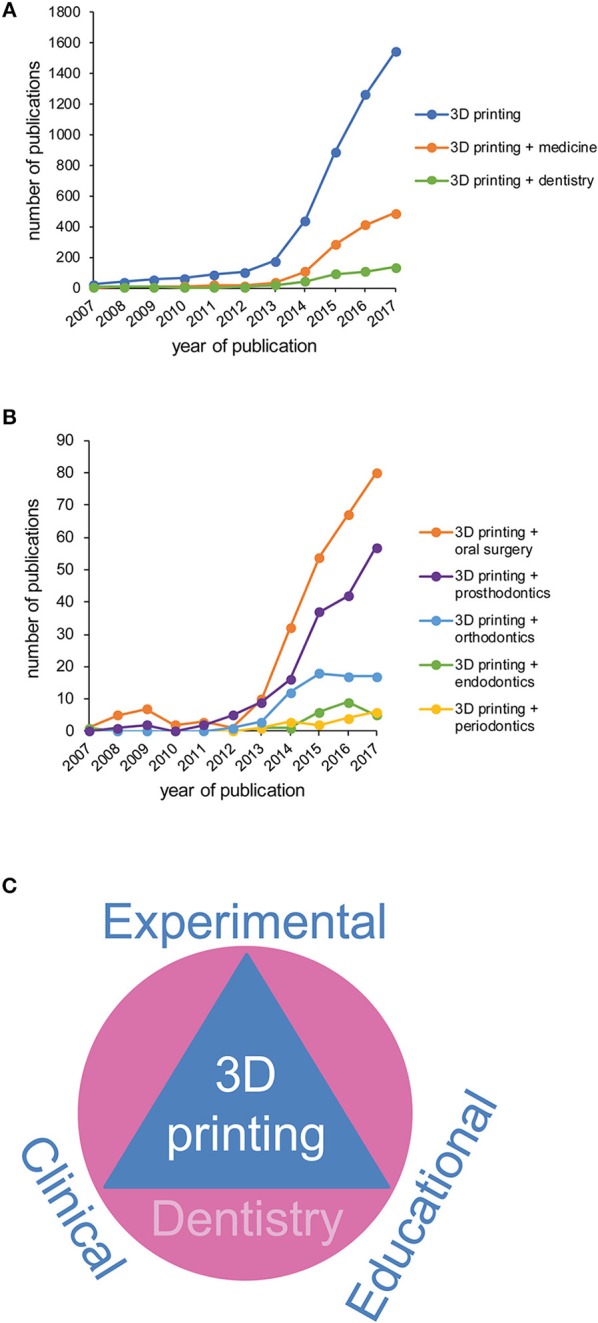
Increasing publication numbers in 3D printing for a variety of dental specialties. **(A)** Number of publications on 3D printing in general and 3D printing in medicine or dentistry in particular (Pubmed.gov; Search date: 01-25-2018; Search algorithm: “3D printing”; “3D printing” AND medicine; “3D printing” AND dentistry) from 2007-2017. **(B)** Number of publications on 3D printing in a variety of dental specialties (Pubmed.gov; Search date: 01-25-2018; Search algorithm: “3D printing” AND “oral surgery”; “3D printing” AND “endodontics”; “3D printing” AND “periodontics”; “3D printing” AND “endodontics”; “3D printing” AND “orthodontics”; “3D printing” AND “prosthodontics”) from 2007-2017. **(C)** Applications for 3D printing in dentistry include experimental, clinical and educational approaches.

In the last few years development of 3D printing for medical and dental applications has increased strikingly. The drive behind advancement in 3D printing for medicine and dentistry emerges from the possibility of individualized products, savings on small scale productions, eased sharing and processing of patient image data and educational upgrading. This trend is reflected by the increasing number of publications on this topic (Figures 1A,B). Publication numbers for 3D printing in general, in medicine and in dentistry in particular increased over the past 10 years in which overall number of publications on 3D printing are higher in medicine than in dentistry (Figure 1A). Looking at the dental specialties it becomes evident that the attention in 3D printing is mainly focused on applications in oral surgery and prosthodontics, followed by orthodontics, while there are limited numbers of publications on applications in periodontics and endodontics (Figure 1B).

**Figure 2:**
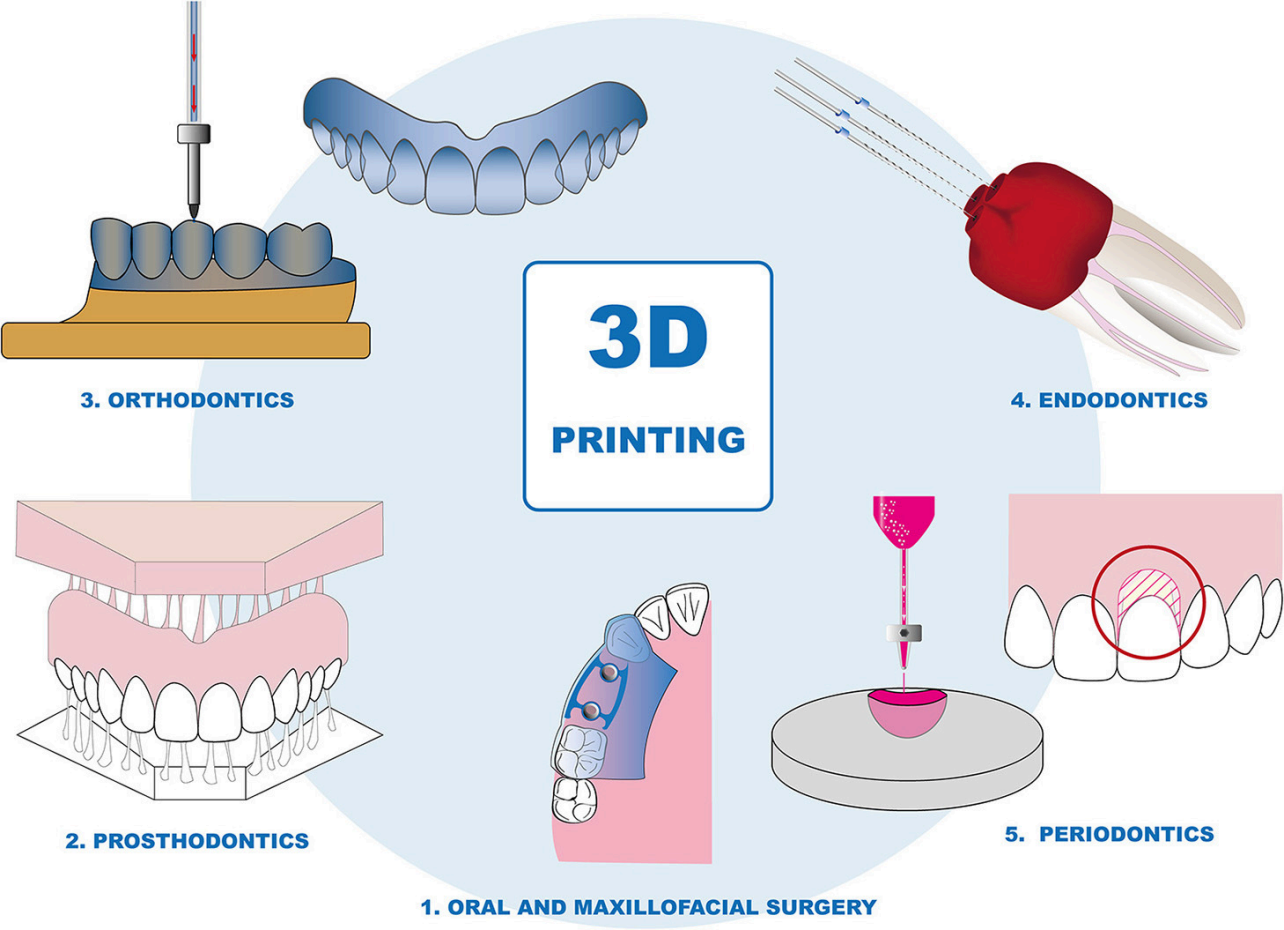
Schematic representation of possible applications of 3D printing in dentistry. Manufacturing anatomical models, guides, and scaffolds for bone defects in oral and maxillofacial surgery; 3D printed dentures and prosthesis in prosthodontics; 3D printed dental models and clear aligners in Orthodontics; Computed tomography based endodontic guides for Root canal treatments; 3D printed scaffolds in Periodontics.

**Figure 3:**
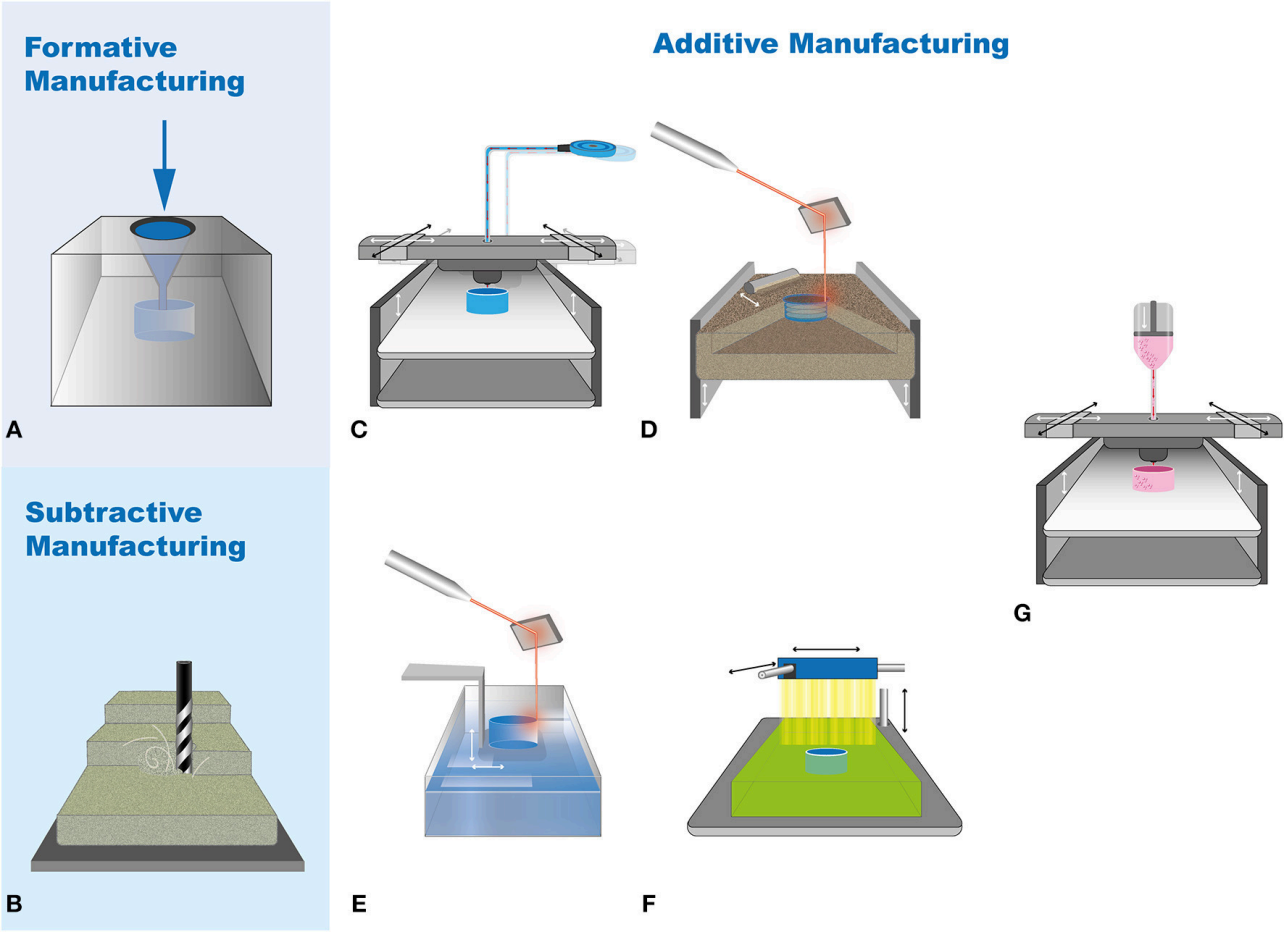
Overview on the different manufacturing approaches. Conventional approaches comprising **(A)** Formative, **(B)** Subtractive manufacturing; widely applied additive manufacturing methods including **(C)** Fused deposition modeling (FDM), **(D)** Selective laser sintering (SLS), **(E)** Stereolithography (SLA), **(F)** Polyjet and **(G)** Bioprinting. Adopted from (Knowlton et al., 2015; Ji and Guvendiren, 2017; Ligon et al., 2017).

Additive manufacturing is gaining rapid potential in nearly all dental fields (Figure 2, Table 1). It differs from formative (Figure 3A) and subtractive manufacturing (Figure 3B) as in the additive manufacturing process the object is “printed” by adding the building material layer by layer (Figures 3C–F). The most widely applied additive manufacturing methods include fused deposition modeling (FDM), selective laser sintering (SLS), stereolithography (SLA), polyjet printing, and bioprinting (Knowlton et al., 2015; Rasperini et al., 2015; Visscher et al., 2016; Ligon et al., 2017; Moroni et al., 2017; Zhang et al., 2017) (Figure 3). Fused deposition modeling (FDM) printers (Figure 3C) are the most common to begin with in a medical or dental set-up owing to its wide availability, moderately reliable printing quality, ease of installation, and use and economic affordability (Huang et al., 2017). It is competent with a number of materials like acrylonitrile butadiene styrene (ABS) and polylactic acid (PLA) (Kalsoom et al., 2018). The spooled material is supplied into a hot nozzle, melting and extruding it in the X-Y dimensions, one layer at a time, before the nozzle is elevated or the print bed drops down (Figure 3C). It is the printer of choice for the in house production of easily accessible anatomical models, but for complex anatomies, the higher printing time, limited color selection, moderate printing resolution and complete removal of the support material are technical limitations (Huang et al., 2017). Both SLS and SLA (Figures 3D,E) use laser to scan and build the object layer-by-layer, while SLS uses powder-based material for printing the object, SLA is based on a liquid resin material (Mazzoli, 2013; Kalsoom et al., 2018). It overcomes the printing resolution and support material limitations of the FDM, however, object shrinkage is a matter of concern. Biocompatible polymeric implants, replication of intricate geometries, and biodegradable scaffolds for tissue engineering are a few of its major applications (Mazzoli, 2013). The printer with highest resolution that is commercially available is the polyjet printer (Figure 3F) where the 3D model is created, one layer at a time, by the printer heads jetting layers of liquid photopolymer onto a build tray, followed by UV light curing (Ionita et al., 2014). The advantages of polyjet printers are a wide choice of printing materials with varieties in density, hardness, flexibility, porosity, resolution as fine as 25 microns, fast printing process, and replication of complex geometries. The disadvantages are the post-print model processing such as intensive washing and removal of support material (Ionita et al., 2014). It finds major application in surgical planning on patient-specific 3D models with complicated geometries, surgical stents and guides, phantoms for orthopedic and cardiac surgeries, and scaffolds for tissue engineering (Klein et al., 1992; Tardieu et al., 2007; Hung et al., 2016; Osman et al., 2017).

**Table 1:** Different types of 3D printers and their potential dental application.

**3D Printer**	**Materials**	**Potential application in dentistry**
Fused Deposition Modeling (FDM)	Thermoplastic polymers such as polylactic acid (PLA), acrylonitrile butadiene styrene (ABS), polycarbonate (PC), polyether ether ketone (PEEK), etc.	In-house production of basic proof-of-concept models, low-cost prototyping of simple anatomical parts
Stereolithography (SLA)	A variety of resins for photopolymerization, ceramic filled resins, etc.	Dental models, surgical guides and splints, orthodontic devices (aligners and retainers), castable crowns, and bridges.
Selective Laser Sintering (SLS)	Powder such as alumide, polyamide, glass-particle filled polyamide, rubber-like polyurethane, etc.	Hospital set up for metal crowns, copings and bridges, metal or resin partial denture frameworks
Polyjet printing	A variety of photopolymers	Hospital set-up manufacturing of craniomaxillofacial implants, sophisticated anatomical models, drilling and cutting guides, facial prosthesis (ear, nose, eye)
Bioprinter	Cell-loaded gels and inks based on collagen, photopolymer resins, agarose, alginate, hyaluronan, chitosan, etc.	Cell-laden scaffolds for hard and soft tissue printing

Following the increasing attention toward these 3D printing methods in the last decade, its utilization in regenerative medicine, tissue engineering, and research has emerged as the most investigated fields of interest. In regenerative medicine, the process of combining cells with 3D-printed polymers for creating 3D cell cultures for tissue engineering (Figure 3G) drug screening or *in vitro* disease models is gaining wide popularity (Zhang et al., 2017). Bioprinting using cell ink-based bioprinters or spheroid/microtissue-based systems has been developed to generate artificial “tissues” and shown to allow the setup of complex 3D *in vitro* models (Blakely et al., 2015; Knowlton et al., 2015; Ip et al., 2016; Ji and Guvendiren, 2017; Zhang et al., 2017; Athirasala et al., 2018). With that, additive manufacturing has given a new face to the discipline of stem cell therapeutics with the flexibility of printing cells into the desired functional 3D complex, employing it for transplantation and regeneration (Murphy and Atala, 2014). A multitude of materials is used to fabricate cell-laden 3D-printed scaffolds, for example, chitosan (Intini et al., 2018), calcium silicate complex (Chen et al., 2018), and controlled-release polymeric materials with bioactive agents (Rahman et al., 2018). 3D printing has found its application in generating optimal volumes of human bone and skin grafts *in vitro* (Lee et al., 2014; Almela et al., 2018). This is gaining immense potential to replace the current strategies of procuring autografts which are associated with donor site morbidity and loss in structure (Chiarello et al., 2013).

The entire process of additive manufacturing technology can basically be divided into four steps: (1) creating a digital 3D model designed with a software or using intraoral scans or computed tomography data. (2) processing and slicing of the 3D model into many two-dimensional layers. (3) printing the 3D end product layer by layer. (4) post-processing of the printed object (Chia and Wu, 2015; Ligon et al., 2017). This basic workflow can be applied for the different printing technologies, using a wide range of materials as polymers, metals or ceramics. We considered multiple reviews available online addressing specific spheres of medical applications as well as techniques and methodologies (Stanek, Manas, Manas, and Navratil; Chae et al., 2015; Choi and Kim, 2015; Farré-Guasch et al., 2015; Torabi et al., 2015; Dodziuk, 2016; Hoang et al., 2016; Shafiee and Atala, 2016; Stansbury and Idacavage, 2016; Tack et al., 2016; Bhargav et al., 2017; Derakhshanfar et al., 2018; Mardis, 2018). In this review we aim to draw together the dental experimental, clinical, and future educational aspects of 3D printing under one roof which has not been done in the past (Figure 1C). This makes it an accessible platform for scientists to budding dentists and dental surgeons in the field of additive manufacturing (Figure 2).

## Oral and maxillofacial surgery

The development of medical 3D imaging generated by computed tomography (CT) has enabled more precise diagnosis and improved treatment planning (Marsh and Vannier, 1983; Cutting et al., 1986). Additive manufacturing has already taken its spot for almost three decades in the field of oral and maxillofacial surgery when anatomical models have been fabricated using stereolithographic methods based on CT data (Klein et al., 1992). Since then, these models have been beneficial for diagnosis, pre-surgical planning, acting as a reference during surgery, and in the manufacturing process of custom implants (Erickson et al., 1999). With the inclusion of these additively manufactured anatomic models into the educational system, the future generation of medical and dental practitioners can avail from the progress in 3D printing. Subsequently, this has led to the development of surgical drilling or cutting guides and more recently to individual bone grafts and scaffolds making 3D printing in oral and maxillofacial surgery an important tool.

### Experimental approaches

Bone grafting is a common practice in reconstructive surgery and employs three types of graft sources: autogenous, autologous, and allogenic. Allogenic grafts as compared to autologous grafts are considered free from ethical, infectious, size-limitation, and donor site morbidity issues. Nevertheless, they lack osteogenic and osteoinductive potential (Hikita et al., 2017). With the introduction of additive manufacturing, it is possible to generate customized implants and scaffolds for bone and tissue regeneration by using biocompatible materials for orofacial defects (Hixon et al., 2017; Tsai et al., 2017; Wurm et al., 2017). Ranging from calcium phosphate biomaterials in the form of hydroxyapatite, β-tricalcium phosphate to polyglycolic acid and polylactic acid and to scaffolds consisting of bioactive magnesium-calcium silicate/ poly-ε-caprolactone, there has been a rapid advancement in the materials used for bone and tissue grafting regeneration by utilizing additive manufacturing (Saijo et al., 2009; Xu et al., 2010; Ciocca et al., 2013; Tsai et al., 2017). With 3D printing it is not only possible to generate tailored scaffolds in the desirable dimensions but also to adjust the properties of these materials with regards to porosity, surface texture, and design. It is possible to add osteoinductive factors, like bone morphogenetic proteins (BMP-2 and BMP-7) for stimulating osteogenic differentiation to increase the integration of bone tissue into the printed scaffolds for better cell adhesion, proliferation, and vascularization (Knippenberg et al., 2006; Sándor et al., 2014; Tsai et al., 2017). Such scaffolds have been tested for cell-free strategies and seeded with stem cells (Wang et al., 2016; Zhou et al., 2016; Yao et al., 2017). *In vitro* and *in vivo* studies have been performed supporting the fabrication of 3D printed titanium and zirconium implants (Wang et al., 2012; Mangano et al., 2014; Anssari Moin et al., 2016). There are, however, no long-term studies to support their clinical application. It would be interesting to see how these materials affect the healing process and osseointegration. In the development of bone bioprinting, much work has been put in the development of feasible bio inks and hydrogels including inks based on decellularized matrix (Wenz et al., 2017; Pacifici et al., 2018). In addition to biocompatibility, these hydrogels have been further functionalized as carriers for growth factors or as gene activated matrices (Miller et al., 2009; Cooper et al., 2010; Cunniffe et al., 2017).

### Clinical approaches

Accurate analysis of the defect using 3D imaging methodologies aids in a more reliable diagnosis (Oh, 2018). Contour models, guides, splints, and implants are the four different categories of three-dimensionally printed surgical objects. Craniofacial anatomical models were the first computer-aided designing and computer-aided manufacturing (CAD/CAM)-based application in oral and maxillofacial surgery. Adapting a milling machine custom-made orthopedic model, Brix and Lambrecht were the first to fabricate anatomical skull models based on CT data in 1987 (Brix and Lambrecht, 1987; Lambrecht and Brix, 1990). Milling machines are limited in case of complex anatomical structures. Therefore, in 1992, Klein *et al*. published a method to produce a model using stereolithography (Klein et al., 1992). Based on that model a maxillary prosthesis was custom made whereas Bill et al. used a 3D printed model for preoperative planning of the surgery, where an allogenic bone transplant was used for cranioplasty (Bill et al., 1995).

3D printing also made it conceivable to completely plan and perform the surgical reconstruction of maxillomandibular defects by three-dimensional virtual techniques with immediate prosthetic loading (van Steenberghe et al., 2005; Wang et al., 2018). Based on the diagnosis, the clinician performs implicitly customized surgical planning for every case using 3D software (van Steenberghe et al., 2005). These anatomic simulations acquaint the surgeon with an intraoperative situation and help him or her to prepare the required instruments and procedures (Jacobs and Lin, 2017). Keeping in mind individual treatment objectives, a simulated model of the final treatment outcome can be fabricated using 3D printing. This helps the patient to understand the surgical strategy and visualize the treatment outcome even before the execution of the surgery (Wilde and Schramm, 2016). Thus, additive manufacturing enables the clinician to obtain the best treatment results and to improve the appearance and quality of life of the patients who undergo facial surgery.

A crucial step in the procedure of digital surgical planning and execution is the designing of surgical guides and templates for improved precision of the operation. They are based on the information obtained by CT imaging and computer software analysis of the maxillomandibular defect (Hu Y. K. et al., 2017). By the use of several commercial software packages it is also possible to yield a digitally planned and printed surgical drilling or cutting guide. It has been shown to have less defects, margin control, and bone compromises. Virtual 3D plan is created on screen to be transferred to the operator site. Thus, it acts as an interface between the virtual plan and the physical patient (Goodacre et al., 2017; Witjes et al., 2018).

3D printing is also applied in the field of orthognathic surgery. A problem that occurs in these procedures is the instability of the condyle and the fossa of the temporomandibular joint, also known as autorotation. This instability makes the correct placement of the maxilla difficult. One approach to solve this problem is the so-called personalized orthognathic surgical guide (POSG) system. The positioning of the bone elements, drill holes for screws and surgical aids is pre-determined by the computer software used and the custom titanium plates can only be placed if the bone segments are exactly in the correct position (Li B. et al., 2017). In addition, patient-specific titanium plates for fixation are manufactured by a 3D printer (Philippe, 2013), providing stability to the construct during the operation (Polley and Figueroa, 2013). Hence, with the help of 3D printed guides, the correct placement of the bone segments is ensured.

### Educational approaches

Along with vast clinical application, 3D printing is the ultimate tool for education and training in oral surgery (Werz et al., 2018). It is expected that very soon, there will be a paradigm shift in the training and educational protocols across the globe. 3D printing offers great opportunities in the field of replicating orofacial anatomy and complex geometry with the highest precision that can be employed to train students and practitioners for performing various maxillofacial operations (Lambrecht et al., 2009). This can be achieved by using high end 3D printers that allow both hard and soft tissues to be replicated in a single training jaw (Yusa et al., 2017). These magnified 3D-printed anatomical models could also help to train students in their three-dimensional spatial orientation and support communication between the clinician and the patient

Thus, there is a strong potential of 3D printing in oral and maxillofacial surgery, not just from a research point of view but also in the clinical and education fronts.

## Prosthodontics

**Figure 4:**
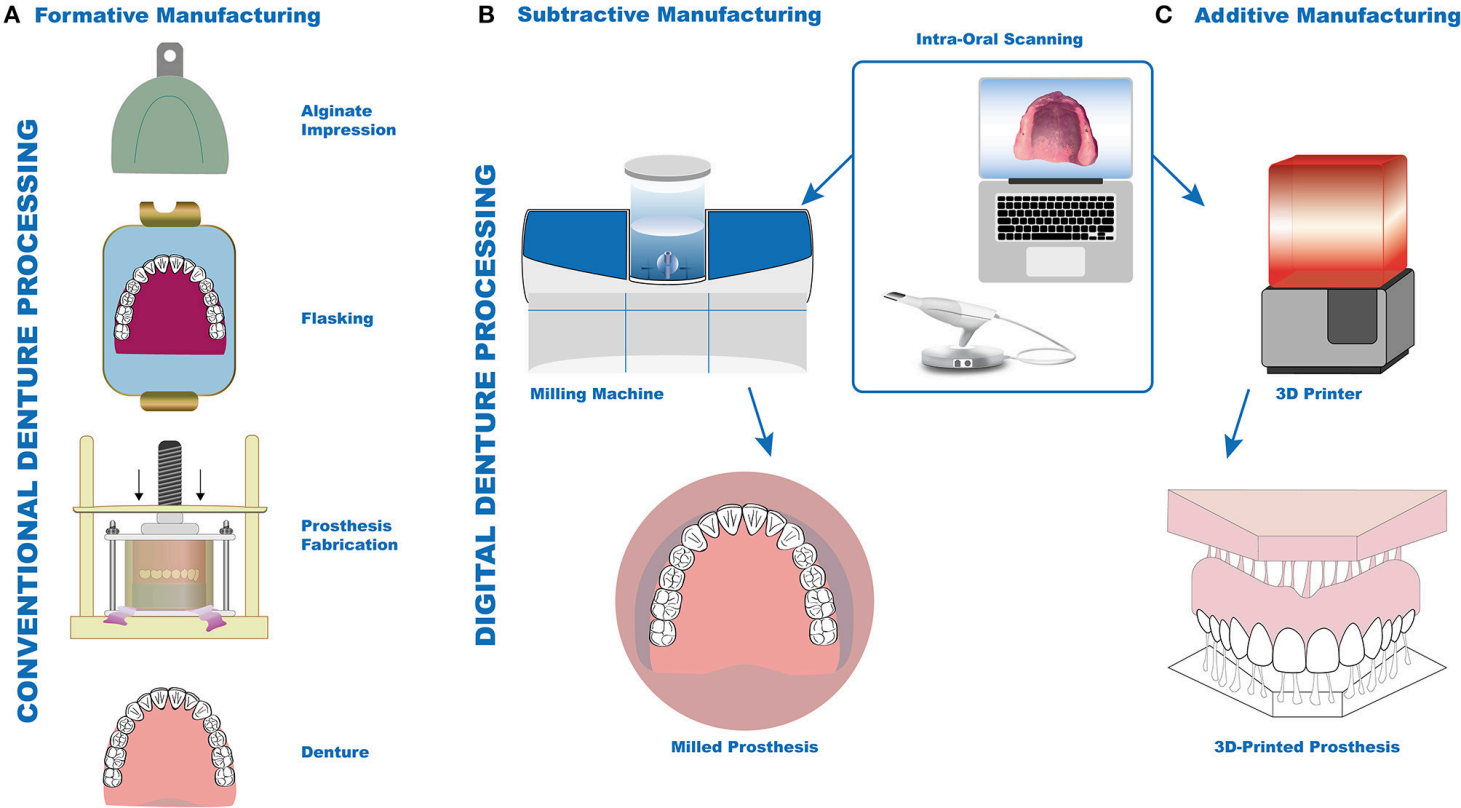
Conventional and digital prosthesis fabrication approaches. Conventional approach for denture fabrication by alginate impression and flasking method (**A**, Formative manufacturing). Digital approach with intra-oral scanning-based impression; manufacturing of denture either by CAD/CAM (**B**, subtractive manufacturing) or 3D printer (**C**, additive manufacturing).

Replacing missing teeth has always been a field of progressive advancement in dentistry, dating back to historic times when materials such as wood, stone, gold, silver, and even extracted teeth from cadavers were used to replace the missing dentition and other parts of the jaw (Freedman, 2011). Traditionally, silicone polymers or alginate were used to produce intraoral impressions and compression- or injection-molding techniques (Figure 4) were used to fabricate dentures (Nogueira et al., 1999). This process is time-consuming, cumbersome and requires a highly skilled dental technician (Yuzbasioglu et al., 2014), especially in case of patients with gag reflex (Hacker et al., 2015), tumor resection, scarred lips post-resection of cancer (Kim et al., 2017), temporomandibular joint defects, or oral deformities. Ongoing research based on additively manufactured materials used to fabricate removable and complete dentures in prosthodontics has shown positive results so far with regards to physical and technical properties (Chen et al., 2015). With progressing advancement in digital workflow it is possible to directly print these prosthesis from silicone providing acceptable esthetics and reducing the number of appointments for the patient at the same time (Unkovskiy et al., 2018). Bioprinting via the production of oral tissue equivalents might help to develop novel models to evaluate the biocompatibility of novel materials and thereby optimize research and development in material science.

### Experimental approaches

Metallic and polymer-based materials are common in additive manufacturing of dental prosthesis and crowns while the use of ceramics is yet to be explored (Ebert et al., 2009). Published *in vitro* studies have shown that ceramics manufactured by lithography where the object is printed layer by layer, show comparable mechanical properties to milled ceramics (Uçar et al., 2018). However, manufacturing process, and strength and fracture toughness are areas that require further research. Most of the 3D printing techniques used today as selective laser sintering, selective laser melting or stereolithography usually result in porous structures whereas ink-jet printing enables production of complex dense ceramic-like structures (Ebert et al., 2009). To improve the mechanical properties of ceramics and increase its homogeneity, porosity should be eradicated resulting in a denser and more compact structure (Uçar et al., 2018). More research is required toward accomplishing the state-of-the-art in ceramics manufactured by 3D printing.

### Clinical approaches

With the introduction of intraoral scanning and 3D printing, denture fabrication has become a more patient friendly procedure (Hu F. et al., 2017). Published case reports indicate that now it is feasible to successfully fabricate removable partial dentures for patients with reduced mouth opening or lip contractures (Kim et al., 2017). Fixed and removable dentures manufactured by 3D printing are clinically acceptable and have physical properties comparable to conventionally fabricated dentures (Gan et al., 2018). Studies have shown that 3D printing can be successfully employed for metal implant prosthesis using selective laser melting and electron beam melting (Revilla León et al., 2017). This leading-edge technology can be employed to reduce the tedious work of a dental technician and provide a more precise framework compared to the conventional framework. Metal crowns and interim resin restorations have shown comparable accuracy and marginal fit with respect to milled restorations (Alharbi et al., 2017). Thus, we see that additive manufacturing has a promising role to play in prosthodontics, especially in patients with facial disabilities or gag reflexes.

### Educational approaches

In the past few years there has been an exemplary shift in the training of dental students and professionals on idealistic plastic typodonts to more real-life 3D-printed models that are based on data obtained by intraoral scans of patients (Hugger et al., 2011). This concept has been utilized in prosthodontics for training dentists on customized real patient-based models for veneer and crown preparation since in the mouth teeth are usually rotated and twisted or contain fillings, which makes the preparation of bridges and crowns more challenging (Kröger et al., 2017). The technique of polyjet printing has successfully been used to create models in different levels of hardness, replicating that of healthy enamel, dentin and caries so that the trainees experience the proprioception of working on a real tooth (Schweiger et al., 2016).

## Orthodontics

3D printing has reformed the era of precision medicine by delivering customized, efficient, highly precise, and reproducible facilities in the field of dentistry, including orthodontics (Jheon et al., 2017). Several years ago Normando et al. introduced the idea of using 3D face scans and 3D printing to print not only the anatomically correct and precise dental arches of patients but also orthodontic brackets (Normando, 2014). As a result, patient-specific adjustments in terms of angulation, bending, and material selection during the manufacture of brackets are possible (Krey et al., 2016). With the help of this computer-aided technique it is now possible to virtually present the changes caused by the braces in advance (Jheon et al., 2017).

### Experimental approaches

Within biomedicine the fundamental understanding of cartilage growth and bone biology is currently being tested in animal models to modify mandibular growth and modulate tooth movement, respectively. Some of these discoveries will ultimately lead to clinical applications in orthodontics for growth modification, accelerating orthodontic tooth movement, and enhancing anchorage or retention of teeth (Jheon et al., 2017). Recently published studies have used the CT data of an adolescent orthodontic patient over a period of 1 year to print 3D models of the mandible using medical imaging software. The data obtained was analyzed for mandibular growth (Reynolds et al., 2011). The results of these studies were in line with human cadaveric and implantable marker studies in the past (Björk and Skieller, 1983). This will aid the pediatric surgeons in conducting craniofacial surgery, orthodontists in comprehending the growth pattern, and the authentication of theoretical growth models (Reynolds et al., 2011). Bioprinting of complex oral tissue like structures can help to reveal the biological responses to forces induced by orthodontic treatments. Thereby these models might serve as alternatives to animal experiments which are currently in use (Liu et al., 2017; Seifi et al., 2017).

### Clinical approaches

To date, 3D printing in orthodontics is used primarily for the production of orthodontic aligners for correcting misaligned teeth. These aligners can be removed at any time by the patient and in most cases they are only worn at night (Dodziuk, 2016). Salmi et al. described the possibility of a so-called rapid tooling method for the production of custom-made, removable regulatory splints, called aligners. These orthodontic aligners can be used on patients with slight malpositioning of the teeth or after fixed orthodontic treatment (Salmi et al., 2012). Using computer software, the teeth are digitally placed in the desired position. After presenting the 3D model, the patient-specific casting mold is created. The mold is printed using the stereolithography method where the product is built up layer by layer during the printing process. From the finished mold, the orthodontic aligner is then casted with silicone (Martorelli et al., 2013).

The use of 3D printing in the manufacturing of splints for a patient with temporomandibular joint dysfunction (TMJ) disorders was also described (Salmi et al., 2013). The occurrence of TMJ in adults is between 25 and 50% and the patients are more likely to suffer from malocclusions, such as cross-bites (Carlsson, 1999). These malocclusions cause excessive signs of wear and tear on the teeth accompanied by pain in the masticatory muscles. Due to the high prevalence of these malalignments in dentition, further research and improvement in the production of splints is desirable (Carlsson et al., 2004). Employing rapid prototyping technology allows improvement in tension of the masticatory muscles. Furthermore, this approach saves time and money and increases precision by reducing the number of manual steps throughout the process (Salmi et al., 2013).

It is known that fixed orthodontic treatment is a time-consuming and expensive procedure. Minimizing the treatment time and the number of appointments is not only favorable for the patient but also precludes tooth demineralization and root resorption (Abella et al., 2018). These new approaches encourage the fabrication of 3D-printed brackets which are the contact point between the wire and the teeth in fixed orthodontics. By digitally planning the tooth movement, designing the brackets tailored to the individual tooth surface and accurately positioning them using 3D-printed guides (Creekmore and Kunik, 1993), it is possible to achieve the preferred treatment outcomes with expedition of the whole procedure. Furthermore, printing of guides for the placement of temporary anchorage devices or indirect bonding guides for the correct positioning of the brackets may play a major role in orthodontics in the future. Also, auxiliary orthodontic devices such as Herbst, Andresen, and sleep apnea appliances can be manufactured by CAD/CAM technology leading to an excellent intraoral fit (Farronato et al., 2011; Al Mortadi et al., 2012). Although clinical studies presented patient satisfaction using this technique, further groundwork is required with regards to the stability and comfortable design of the 3D-printed orthodontic brackets.

### Educational approaches

The importance of 3D documentation in orthodontic and craniofacial disorders has been endorsed since the last decade. Plaster models have now been replaced by digital information and data (Rischen et al., 2013). This does not only dissolve the bulk storage problems often faced by orthodontists, but also opens a new horizon of education and research. Rescuing the patient from repeated exposure to ionizing radiation, 3D-printed models have been used to establish new theorems and relationships between alveolar area and the need for extraction (Konvalinkova et al., 2018). In the future it will be possible to use real patient additively manufactured dental models based on intraoral scans or cone beam computed tomography (CBCT) for training fixed and removable orthodontics to dental students.

## Endodontics

As seen in the above described fields of dentistry, 3D printing has also carved a prolific niche in the endodontic discipline as well (Anderson et al., 2018). The paradigm shift from manual to digital workflow in endodontics has given rise to an unmatched streamlining of the procedure, greater precision and accuracy, ameliorating patient comfort, a breakthrough in regenerative endodontics and advancing the operator skills by training and education (Shah and Chong, 2018).

### Experimental approaches

Additive manufacturing has invaded the field of experimental regenerative endodontics by its capability to preserve the natural tooth rather than replacing it by prosthetic surgery (Murray et al., 2007). The principle of 3D printing can be applied to deliver stem cells, pulp scaffolds, injectable calcium phosphates, growth factors, and for gene therapy in the endodontics (Murray et al., 2007). Various types of calcium phosphate cements have been developed by 3D printing to form porous scaffolds for regeneration of the pulp-dentin complex (Xu et al., 2017). Research has shown that application of 3D-printed polycaprolactone coated with freeze-dried platelet-rich plasma to the dental pulp cells has an improved osteogenic activity *in vitro* (Li J. et al., 2017). Also anatomically shaped tooth-like tissue has been generated using 3D printed poly-epsilon-caprolactone and hydroxyapatite scaffolds (Kim et al., 2010). Also, bioprinting approaches were developed using dentin-derived bionics. For scaffold-free approaches dental pulp cell-derived spheroids have shown promising results for regenerative strategies (Xiao and Tsutsui, 2013; Dissanayaka et al., 2014, 2015; Neunzehn et al., 2014; Janjić et al., 2018).

### Clinical approaches

Clinically, additive manufacturing in endodontics finds application in guided apicoectomy and endodontic access cavity preparation. Published studies have shown the efficacy and advantages of guided access cavity preparation over the conventional one. 3D-printed guides can be a useful time-saving aid in calcified canal cases and apical periodontitis (Connert et al., 2018). Endodontic procedures are quite challenging in teeth with anomalies in root canal anatomy making access cavity preparation, disinfection, and obturation a tough procedure (Byun et al., 2015). Published case reports have shown the potential role of 3D printing in this field by making additively manufactured tooth models with internal root canal structures that can be used as a base to print a guide for the endodontic treatment of such challenging cases (Byun et al., 2015). Furthermore this technique can be applied to molars with complex root canal anatomies since the radiograph only gives 2D information of the root canal, often obliterating the accessory and lateral canals (Rodrigues et al., 2016).

### Educational approaches

Additive manufacturing has a major role in endodontic training and education. There has been an increasing trend in many dental schools across the world toward replacing typodont teeth that are known to have idealistic root canal anatomies with 3D-printed tooth models, based on computerized-tomographic images of extracted teeth with more realistic anatomical root canal structure (Byun et al., 2015). 3D-printed models and computer software such as haptic simulators aid in the development of endodontic skills by providing visual, acoustical, and tactile proprioception to the user. Critical anatomical structures like nerves and blood vessels or thick cortical bone covering root apices often lead to procedural errors where these models can serve as a boon to prepare the surgeon for challenging situations (Shah and Chong, 2018). Thus, we see that 3D printing has a very promising role and future advancement in surgical and non-surgical endodontics.

## Periodontics

Another area of dentistry in which 3D printing is used is periodontology with the focus being on regenerative periodontology in research and 3D-printed guides for esthetic gingival correction. The periodontium is a complex tissue system consisting of several components like bone, gingiva, and cementum. Each tissue has different properties and tissue regeneration in the oral cavity is accordingly controlled by several cell types, signaling mechanisms and interactions.

### Experimental approaches

The term additive biomanufacturing, signifying the application of 3D printing (Hoang et al., 2016) in manufacturing 3D-printed scaffolds to support tissue regeneration in a defect (Hung et al., 2016) is popularly used in periodontics. Bone and tissue loss accompany periodontitis and the concept behind using this technology is to restore the resorbed periodontal tissue and bone deficiencies by supplying the surrounding tissue with growth factors, genetically modified cells, or bioactive proteins over a certain period of time (Larsson et al., 2016). However, damage to the periodontal tissue can also lead to difficulties with the implant placement or cause implant loss as the remaining tissue does not provide sufficient support for osseointegration. Here again, 3D printing finds its application in the procedure called guided tissue regeneration. The principle of controlled tissue regeneration is to prevent the ingrowth of rapidly regenerating tissues such as the oral epithelium into the defect and at the same time provide room to the slow-growing bone tissue for regeneration (Carter et al., 2017). Advancements in 3D-printed membrane structure, improving its integrity and function in the oral cavity, making it more resistant to the occlusal forces, are being effectuated (Bottino et al., 2017).

Various 3D printing techniques find application in tissue regeneration based on the requirements of the defect area. A CT scan of the defect in a patient serves as template for the creation of 3D objects. Based on the CT image, a printed wax mold is designed for the production of a scaffold that can be used to improve the immigration of periodontal ligament cells, which are responsible for the connection of dental cementum and tooth root (Pilipchuk et al., 2016). Improved regeneration of the alveolar tissue using 3D polycaprolactone (PCL) scaffolds has been shown (Li J. et al., 2017). With the invention of 3D-printed biphasic scaffolds, it is now possible to utilize and guide multiple periodontal cell types during the healing process. In *in vivo* investigations in mice it has been noted that the biphasic frameworks have advantages over scaffolds which are produced without the exact specification of a printed mold. The used method provided predictable orientation, improved organization of the periodontal ligament, and controlled tissue infiltration. Complex clinical cases have been reported where individualized 3D-printed scaffolds have been applied for periodontal regeneration (Rasperini et al., 2015). Studies on bioprinting of periodontal cells in hydrogels have proven the feasibility of the technology *in vitro* (Ma et al., 2015; Xu and Hu, 2017). However, in addition to tissue engineering strategies, the technology can also be applied for other purposes. Bioprinting of 3D arrays of hydrogels loaded with periodontal stem cells was used as *in vitro* model to evaluate the impact of extra cellular matrix. Similar complex *in vitro* models can be developed as screening assays for novel target for periodontal regeneration and the optimization of biomaterials (Xu and Hu, 2017). Also scaffold-free approaches of bioprinting seem feasible as spheroids (Janjić et al., 2017; Kurzmann et al., 2017) and more complex microtissues (Janjić et al., 2017) have been generated successfully from periodontal ligament and gingival cells. The application of such self-assembled building blocks for periodontal regeneration has been proposed (Yang et al., 2010; Berahim et al., 2011).

### Clinical approaches

Clinically, 3D printing has gained popularity in gingival esthetic surgeries in the anterior region of the oral cavity (Li Z. et al., 2017). Patient specific surgical guides are printed and used for gingivectomy procedures and smile designing. Such templates are known for their accuracy, customization, and precision. Educational and training models based on computerized tomographic scans of patients are being increasingly developed for the gaining of better surgical skills (Werz et al., 2018). Through the use of individualized products, advantages over conventional methods can be created in the area of periodontal tissue regeneration and surgeries.

### Educational approaches

In the past, dental students have been trained either on manikins, dental models, or directly on patients for periodontal examination, scoring, and indexing procedures (Heym et al., 2016). Students have often found difficulties, leading to patient discomfort such as pain and bleeding while probing the patients for examination (Heym et al., 2016). Hence, it would be a good approach to print 3D models simulating gums, periodontal tissues, and defects with respective tissue characteristics to develop the correct proprioception and skill before operating the patient. Additive manufacturing also encourages printing models of patients with gingival esthetic defects to be trained using these models and preventing procedural errors (Li Z. et al., 2017).

## Conclusion and future perspective

3D printing has the capacity to revolutionize dentistry. The different technologies have been applied for a variety of purposes in the field of dentistry (Figures 2,3, Table 1). Currently the main focus is on surgical planning and the indirect production of implants or orthodontic aligners by printing the molds for these objects. In addition, 3D printing is used to create personalized tissue engineering scaffolds for usage in oral surgery. Experimental approaches include the application of 3D printing for the production of scaffolds which serve as carriers for growth factors or other bioactive molecules as well as cells. However, the results of previous studies show that 3D printing has many advantages, be it in the fabrication of fixation splints in oral surgery or in orthodontic orthosis molds. Because the print object is produced according to the image of the patient, the print can be tailored to optimally fit the anatomical conditions and thereby accuracy of aligners or implants can be improved.

When selecting the appropriate printing system, account must be taken of material availability, medical properties of the material, time required, and the desired resolution of the print object. The problem that requires further research is the limitation of the available material assortment in particular when moving beyond the canonical polymers as well as the improvement of printing speed and post processing requires. The used materials must meet the dental and technical requirements and biocompatibility standards. It is therefore of great interest to establish new, printable materials for dentistry that meet these requirements, as the expansion of the material range also opens up new possibilities for clinical applications of 3D printing in dentistry.

3D printing has a high potential for education as witnessed above in all the major disciplines of dentistry. It gives the surgeon a better subjective perception of the bone and teeth as compared to the stereotype typodont or acrylic models. With the advancement in materials and technology, the flexibility to manipulate the physical characteristics of additively manufactured materials, the trainees have the opportunity to develop better operative and proprioceptive skills (Hugger et al., 2011; Torres et al., 2011; Werz et al., 2018). Overall, 3D printing-based technologies have a tremendous potential to transform research, treatment methodology, and educational streams of dentistry ameliorating oral health care.

## Author contributions

GO and SN were involved in study design, literature research, data analysis, writing the manuscript. ME, KJ, and AM were involved in the study design, study and manuscript preparation. HA was involved in study design, data analysis, writing, and submission of the manuscript.

### Conflict of interest statement

The authors declare that the research was conducted in the absence of any commercial or financial relationships that could be construed as a potential conflict of interest.

## References

[B1] Abella F. Ribas F. Roig M. González Sánchez J. A. Durán-Sindreu F. (2018). Outcome of autotransplantation of mature third molars using 3-dimensional-printed guiding templates and donor tooth replicas. J. Endod. 44, 1567–1574. 10.1016/j.joen.2018.07.007 30154002

[B2] Al Mortadi N. Eggbeer D. Lewis J. Williams R. J. (2012). CAD/CAM/AM applications in the manufacture of dental appliances. Am. J. Orthod. Dentofacial Orthop. 142, 727–733. 10.1016/j.ajodo.2012.04.023 23116514

[B3] Alharbi N. Alharbi S. Cuijpers V. M. J. I. Osman R. B. Wismeijer D. (2017). Three-dimensional evaluation of marginal and internal fit of 3D-printed interim restorations fabricated on different finish line designs. J. Prosthodont. Res. 62, 218–226. 10.1016/j.jpor.2017.09.002 29032176

[B4] Almela T. Al-Sahaf S. Brook I. M. Khoshroo K. Rasoulianboroujeni M. Fahimipour F.. (2018). 3D printed tissue engineered model for bone invasion of oral cancer. Tissue Cell 52, 71–77. 10.1016/j.tice.2018.03.009 29857831

[B5] Anderson J. Wealleans J. Ray J. (2018). Endodontic applications of 3D printing. Int. Endod. J. 51, 1005–1018. 10.1111/iej.12917 29486052

[B6] Anssari Moin D. Derksen W. Verweij J. P. van Merkesteyn R. Wismeijer D. (2016). A novel approach for computer-assisted template-guided autotransplantation of teeth with custom 3D designed/printed surgical tooling. An *ex vivo* proof of concept. J. Oral Maxillofac. Surg. 74, 895–902. 10.1016/j.joms.2016.01.033 26907556

[B7] Athirasala A. Tahayeri A. Thrivikraman G. França C. M. Monteiro N. Tran V.. (2018). A dentin-derived hydrogel bioink for 3D bioprinting of cell laden scaffolds for regenerative dentistry. Biofabrication 10:024101. 10.1088/1758-5090/aa9b4e 29320372 PMC5796756

[B8] Berahim Z. Moharamzadeh K. Rawlinson A. Jowett A. K. (2011). Biologic interaction of three-dimensional periodontal fibroblast spheroids with collagen-based and synthetic membranes. J. Periodontol. 82, 790–797. 10.1902/jop.2010.100533 21080786

[B9] Bhargav A. Sanjairaj V. Rosa V. Feng L. W. Fuh Yh J. (2017). Applications of additive manufacturing in dentistry: a review. J. Biomed. Mater. Res. B Appl. Biomater. 106, 2058–2064. 10.1002/jbm.b.33961 28736923

[B10] Bill J. S. Reuther J. F. Dittmann W. Kübler N. Meier J. L. Pistner H.. (1995). Stereolithography in oral and maxillofacial operation planning. Int. J. Oral Maxillofac. Surg. 24, 98–103. 10.1016/S0901-5027(05)80869-0 7782651

[B11] Björk A. Skieller V. (1983). Normal and abnormal growth of the mandible. A synthesis of longitudinal cephalometric implant studies over a period of 25 years. Eur. J. Orthod. 5, 1–46. 10.1093/ejo/5.1.1 6572593

[B12] Blakely A. M. Manning K. L. Tripathi A. Morgan J. R. (2015). Bio-Pick, place, and perfuse: a new instrument for three-dimensional tissue engineering. Tissue Eng. Part C Methods 21, 737–746. 10.1089/ten.tec.2014.0439 25530515 PMC4499775

[B13] Bottino M. C. Pankajakshan D. Nör J. E. (2017). Advanced scaffolds for dental pulp and periodontal regeneration. Dent. Clin. North Am. 61, 689–711. 10.1016/j.cden.2017.06.009 28886764 PMC5657339

[B14] Brix F. Lambrecht J. T. (1987). [Preparation of individual skull models based on computed tomographic information]. Fortschr. Kiefer. Gesichtschir. 32, 74–77. 3476440

[B15] Byun C. Kim C. Cho S. Baek S. H. Kim G. Kim S. G.. (2015). Endodontic treatment of an anomalous anterior tooth with the aid of a 3-dimensional printed physical tooth model. J. Endod. 41, 961–965. 10.1016/j.joen.2015.01.016 25732403

[B16] Carlsson G. E. (1999). Epidemiology and treatment need for temporomandibular disorders. J. Orofac. Pain 13, 232–237. 10823035

[B17] Carlsson G. E. Magnusson T. Egermark I. (2004). Prediction of demand for treatment of temporomandibular disorders based on a 20-year follow-up study. J. Oral Rehabil. 31, 511–517. 10.1111/j.1365-2842.2004.01275.x 15189306

[B18] Carter S.-S. D. Costa P. F. Vaquette C. Ivanovski S. Hutmacher D. W. Malda J. (2017). Additive biomanufacturing: an advanced approach for periodontal tissue regeneration. Ann. Biomed. Eng. 45, 12–22. 10.1007/s10439-016-1687-2 27473707 PMC5215138

[B19] Chae M. P. Rozen W. M. McMenamin P. G. Findlay M. W. Spychal R. T. Hunter-Smith D. J. (2015). Emerging applications of bedside 3D printing in plastic surgery. Front. Surg. 2:25. 10.3389/fsurg.2015.00025 26137465 PMC4468745

[B20] Chen J. Ahmad R. Suenaga H. Li W. Sasaki K. Swain M. (2015). Shape optimization for additive manufacturing of removable partial dentures–a new paradigm for prosthetic CAD/CAM. PLoS ONE 10:e0132552 10.1371/journal.pone.0132552 26161878 PMC4498620

[B21] Chen Y.-W. Shen Y.-F. Ho C.-C. Yu J. Wu Y.-H. A. Wang K.. (2018). Osteogenic and angiogenic potentials of the cell-laden hydrogel/mussel-inspired calcium silicate complex hierarchical porous scaffold fabricated by 3D bioprinting. Mater. Sci. Eng. C Mater. Biol. Appl. 91, 679–687. 10.1016/j.msec.2018.06.005 30033302

[B22] Chia H. N. Wu B. M. (2015). Recent advances in 3D printing of biomaterials. J. Biol. Eng. 9:4. 10.1186/s13036-015-0001-4 25866560 PMC4392469

[B23] Chiarello E. Cadossi M. Tedesco G. Capra P. Calamelli C. Shehu A.. (2013). Autograft, allograft and bone substitutes in reconstructive orthopedic surgery. Aging Clin. Exp. Res. 25(Suppl 1), S101–S103. 10.1007/s40520-013-0088-8 24046051

[B24] Choi J. W. Kim N. (2015). Clinical application of three-dimensional printing technology in craniofacial plastic surgery. Arch. Plast. Surg. 42, 267–277. 10.5999/aps.2015.42.3.267 26015880 PMC4439584

[B25] Ciocca L. Donati D. Ragazzini S. Dozza B. Rossi F. Fantini M.. (2013). Mesenchymal stem cells and platelet gel improve bone deposition within CAD-CAM custom-made ceramic HA scaffolds for condyle substitution. Biomed Res. Int. 2013:549762. 10.1155/2013/549762 24073409 PMC3773948

[B26] Connert T. Zehnder M. S. Amato M. Weiger R. Kühl S. Krastl G. (2018). Microguided endodontics: a method to achieve minimally invasive access cavity preparation and root canal location in mandibular incisors using a novel computer-guided technique. Int. Endod. J. 51, 247–255. 10.1111/iej.12809 28665514

[B27] Cooper G. M. Miller E. D. Decesare G. E. Usas A. Lensie E. L. Bykowski M. R.. (2010). Inkjet-based biopatterning of bone morphogenetic protein-2 to spatially control calvarial bone formation. Tissue Eng. Part A 16, 1749–1759. 10.1089/ten.tea.2009.0650 20028232 PMC2952127

[B28] Creekmore T. D. Kunik R. L. (1993). Straight wire: the next generation. Am. J. Orthod. Dentofacial Orthop. 104, 8–20. 10.1016/0889-5406(93)70023-H 8257493

[B29] Cunniffe G. M. Gonzalez-Fernandez T. Daly A. Sathy B. N. Jeon O. Alsberg E.. (2017). ^*^Three-dimensional bioprinting of polycaprolactone reinforced gene activated bioinks for bone tissue engineering. Tissue Eng. Part A 23, 891–900. 10.1089/ten.tea.2016.0498 28806146

[B30] Cutting C. Bookstein F. L. Grayson B. Fellingham L. McCarthy J. G. (1986). Three-dimensional computer-assisted design of craniofacial surgical procedures: optimization and interaction with cephalometric and CT-based models. Plast. Reconstr. Surg. 77, 877–887. 10.1097/00006534-198606000-00001 3714886

[B31] Derakhshanfar S. Mbeleck R. Xu K. Zhang X. Zhong W. Xing M. (2018). 3D bioprinting for biomedical devices and tissue engineering: a review of recent trends and advances. Bioact. Mater. 3, 144–156. 10.1016/j.bioactmat.2017.11.008 29744452 PMC5935777

[B32] Dissanayaka W. L. Zhu L. Hargreaves K. M. Jin L. Zhang C. (2014). Scaffold-free prevascularized microtissue spheroids for pulp regeneration. J. Dent. Res. 93, 1296–1303. 10.1177/0022034514550040 25201919 PMC4462805

[B33] Dissanayaka W. L. Zhu L. Hargreaves K. M. Jin L. Zhang C. (2015). *In vitro* analysis of scaffold-free prevascularized microtissue spheroids containing human dental pulp cells and endothelial cells. J. Endod. 41, 663–670. 10.1016/j.joen.2014.12.017 25687363

[B34] Dodziuk H. (2016). Applications of 3D printing in healthcare. Kardiochir. Torakochirurgia Pol. 13, 283–293. 10.5114/kitp.2016.62625 27785150 PMC5071603

[B35] Ebert J. Ozkol E. Zeichner A. Uibel K. Weiss O. Koops U.. (2009). Direct inkjet printing of dental prostheses made of zirconia. J. Dent. Res. 88, 673–676. 10.1177/0022034509339988 19641157

[B36] Erickson D. M. Chance D. Schmitt S. Mathis J. (1999). An opinion survey of reported benefits from the use of stereolithographic models. J. Oral Maxillofac. Surg. 57, 1040–1043. 10.1016/S0278-2391(99)90322-1 10484104

[B37] Farré-Guasch E. Wolff J. Helder M. N. Schulten E. A. J. M. Forouzanfar T. Klein-Nulend J. (2015). Application of additive manufacturing in oral and maxillofacial surgery. J. Oral Maxillofac. Surg. 73, 2408–2418. 10.1016/j.joms.2015.04.019 25966454

[B38] Farronato G. Santamaria G. Cressoni P. Falzone D. Colombo M. (2011). The digital-titanium Herbst. J. Clin. Orthod. 45, 263–7; quiz 287. 21785192

[B39] Freedman G. A. (2011). Contemporary Esthetic Dentistry-E-Book. Toronto, ON: Elsevier Health Sciences.

[B40] Gan N. Ruan Y. Sun J. Xiong Y. Jiao T. (2018). Comparison of adaptation between the major connectors fabricated from intraoral digital impressions and extraoral digital impressions. Sci. Rep. 8:529. 10.1038/s41598-017-17839-4 29323129 PMC5765160

[B41] Goodacre B. J. Swamidass R. S. Lozada J. Al-Ardah A. Sahl E. (2017). A 3D-printed guide for lateral approach sinus grafting: A dental technique. J. Prosthet. Dent. 119, 897–901. 10.1016/j.prosdent.2017.07.014 29150131

[B42] Hacker T. Heydecke G. Reissmann D. R. (2015). Impact of procedures during prosthodontic treatment on patients' perceived burdens. J. Dent. 43, 51–57. 10.1016/j.jdent.2014.10.013 25446242

[B43] Heym R. Krause S. Hennessen T. Pitchika V. Ern C. Hickel R. (2016). A new model for training in periodontal examinations using manikins. J. Dent. Educ. 80, 1422–1429. 27934667

[B44] Hikita A. Chung U.-I. Hoshi K. Takato T. (2017). Bone regenerative medicine in oral and maxillofacial region using a three-dimensional printer. Tissue Eng. Part A 23, 515–521. 10.1089/ten.tea.2016.0543 28351222

[B45] Hixon K. R. Melvin A. M. Lin A. Y. Hall A. F. Sell S. A. (2017). Cryogel scaffolds from patient-specific 3D-printed molds for personalized tissue-engineered bone regeneration in pediatric cleft-craniofacial defects. J. Biomater. Appl. 32, 598–611. 10.1177/0885328217734824 28980856

[B46] Hoang D. Perrault D. Stevanovic M. Ghiassi A. (2016). Surgical applications of three-dimensional printing: a review of the current literature and how to get started. Ann. Transl. Med. 4:456. 10.21037/atm.2016.12.18 28090512 PMC5220021

[B47] Hu F. Pei Z. Wen Y. (2017). Using intraoral scanning technology for three-dimensional printing of kennedy class i removable partial denture metal framework: a clinical report. J. Prosthodont. 1–4 10.1111/jopr.12712 29143451

[B48] Hu Y. K. Xie Q. Y. Yang C. Xu G. Z. (2017). Computer-designed surgical guide template compared with free-hand operation for mesiodens extraction in premaxilla using “trapdoor” method. Medicine 96:e7310. 10.1097/MD.0000000000007310 28658139 PMC5500061

[B49] Huang Y. Zhang X.-F. Gao G. Yonezawa T. Cui X. (2017). 3D bioprinting and the current applications in tissue engineering. Biotechnol. J. 12:1600734. 10.1002/biot.201600734 28675678

[B50] Hugger A. Hugger S. Kordass B. (2011). [Dental education in Germany: new concepts for the dental curriculum]. Bundesgesundheitsblatt Gesundheitsforschung Gesundheitsschutz 54, 1046–1051. 10.1007/s00103-011-1328-8 21887618

[B51] Hung K.-C. Tseng C.-S. Dai L.-G. Hsu S. (2016). Water-based polyurethane 3D printed scaffolds with controlled release function for customized cartilage tissue engineering. Biomaterials 83, 156–168. 10.1016/j.biomaterials.2016.01.019 26774563

[B52] Intini C. Elviri L. Cabral J. Mros S. Bergonzi C. Bianchera A.. (2018). 3D-printed chitosan-based scaffolds: an *in vitro* study of human skin cell growth and an *in-vivo* wound healing evaluation in experimental diabetes in rats. Carbohydr. Polym. 199, 593–602. 10.1016/j.carbpol.2018.07.057 30143167

[B53] Ionita C. N. Mokin M. Varble N. Bednarek D. R. Xiang J. Snyder K. V.. (2014). Challenges and limitations of patient-specific vascular phantom fabrication using 3D Polyjet printing. Proc. SPIE 9038:90380M. 10.1117/12.2042266 25300886 PMC4188370

[B54] Ip B. C. Cui F. Tripathi A. Morgan J. R. (2016). The bio-gripper: a fluid-driven micro-manipulator of living tissue constructs for additive bio-manufacturing. Biofabrication 8:025015. 10.1088/1758-5090/8/2/025015 27221320

[B55] Jacobs C. A. Lin A. Y. (2017). A new classification of three-dimensional printing technologies: systematic review of three-dimensional printing for patient-specific craniomaxillofacial surgery. Plast. Reconstr. Surg. 139, 1211–1220. 10.1097/PRS.0000000000003232 28445375

[B56] Janjić K. Kurzmann C. Moritz A. Agis H. (2017). Expression of circadian core clock genes in fibroblasts of human gingiva and periodontal ligament is modulated by L-Mimosine and hypoxia in monolayer and spheroid cultures. Arch. Oral Biol. 79, 95–99. 10.1016/j.archoralbio.2017.03.007 28350992

[B57] Janjić K. Lilaj B. Moritz A. Agis H. (2018). Formation of spheroids by dental pulp cells in the presence of hypoxia and hypoxia mimetic agents. Int. Endod. J. 51(Suppl 2), e146–e156. 10.1111/iej.12806 28656722

[B58] Jheon A. H. Oberoi S. Solem R. C. Kapila S. (2017). Moving towards precision orthodontics: an evolving paradigm shift in the planning and delivery of customized orthodontic therapy. Orthod Craniofac Res. 20(Suppl 1), 106–113. 10.1111/ocr.12171 28643930

[B59] Ji S. Guvendiren M. (2017). Recent advances in bioink design for 3D bioprinting of tissues and organs. Front. Bioeng. Biotechnol. 5:23. 10.3389/fbioe.2017.00023 28424770 PMC5380738

[B60] Kalsoom U. Hasan C. K. Tedone L. Desire C. T. Li F. Breadmore M. C. (2018). A low-cost passive sampling device with integrated porous membrane produced using multi-material 3D printing. Anal. Chem. 90, 12081–12089. 10.1021/acs.analchem.8b02893. 30222326

[B61] Kim J.-E. Kim N.-H. Shim J.-S. (2017). Fabrication of a complete, removable dental prosthesis from a digital intraoral impression for a patient with an excessively tight reconstructed lip after oral cancer treatment: a clinical report. J. Prosthet. Dent. 117, 205–208. 10.1016/j.prosdent.2016.07.001 27646801

[B62] Kim K. Lee C. H. Kim B. K. Mao J. J. (2010). Anatomically shaped tooth and periodontal regeneration by cell homing. J. Dent. Res. 89, 842–847. 10.1177/0022034510370803 20448245 PMC3318060

[B63] Klein H. M. Schneider W. Nawrath J. Gernot T. Voy E. D. Krasny R. (1992). [Stereolithographic model construction based on 3-dimensional reconstructed CT sectional image sequences]. Rofo 156, 429–432. 10.1055/s-2008-1032915 1596544

[B64] Knippenberg M. Helder M. N. Zandieh Doulabi B. Wuisman P. I. J. M. Klein-Nulend J. (2006). Osteogenesis versus chondrogenesis by BMP-2 and BMP-7 in adipose stem cells. Biochem. Biophys. Res. Commun. 342, 902–908. 10.1016/j.bbrc.2006.02.052 16500625

[B65] Knowlton S. Onal S. Yu C. H. Zhao J. J. Tasoglu S. (2015). Bioprinting for cancer research. Trends Biotechnol. 33, 504–513. 10.1016/j.tibtech.2015.06.007 26216543

[B66] Konvalinkova M. Urbanova W. Langova K. Kotova M. (2018). Using 3D digital models to establish alveolar morphotype. Folia Morphol. 77, 536–542. 10.5603/FM.a2018.0014 29399755

[B67] Krey K.-F. Darkazanly N. Kühnert R. Ruge S. (2016). 3D-printed orthodontic brackets - proof of concept. Int. J. Comput. Dent. 19, 351–362. 28008431

[B68] Kröger E. Dekiff M. Dirksen D. (2017). 3D printed simulation models based on real patient situations for hands-on practice. Eur. J. Dent. Educ. 21, e119–e125. 10.1111/eje.12229 27470072

[B69] Kurzmann C. Janjić K. Shokoohi-Tabrizi H. Edelmayer M. Pensch M. Moritz A.. (2017). Evaluation of resins for stereolithographic 3D-printed surgical guides: the response of L929 cells and human gingival fibroblasts. Biomed Res. Int. 2017:4057612. 10.1155/2017/4057612 29201905 PMC5671678

[B70] Lambrecht J. T. Berndt D. C. Schumacher R. Zehnder M. (2009). Generation of three-dimensional prototype models based on cone beam computed tomography. Int. J. Comput. Assist. Radiol. Surg. 4, 175–180. 10.1007/s11548-008-0275-9 20033617

[B71] Lambrecht J. T. Brix F. (1990). Individual skull model fabrication for craniofacial surgery. Cleft Palate J. 27, 382–385; discussion 386. 10.1597/1545-1569(1990)027<0382:ISMFFC>2.3.CO;2 2253385

[B72] Larsson L. Decker A. M. Nibali L. Pilipchuk S. P. Berglundh T. Giannobile W. V. (2016). Regenerative medicine for periodontal and peri-implant diseases. J. Dent. Res. 95, 255–266. 10.1177/0022034515618887 26608580 PMC4766955

[B73] Lee V. Singh G. Trasatti J. P. Bjornsson C. Xu X. Tran T. N.. (2014). Design and fabrication of human skin by three-dimensional bioprinting. Tissue Eng. Part C Methods 20, 473–484. 10.1089/ten.tec.2013.0335 24188635 PMC4024844

[B74] Li B. Shen S. Jiang W. Li J. Jiang T. Xia J. J.. (2017). A new approach of splint-less orthognathic surgery using a personalized orthognathic surgical guide system: a preliminary study. Int. J. Oral Maxillofac. Surg. 46, 1298–1305. 10.1016/j.ijom.2017.03.025 28552440 PMC5663459

[B75] Li J. Chen M. Wei X. Hao Y. Wang J. (2017). Evaluation of 3D-printed polycaprolactone scaffolds coated with freeze-dried platelet-rich plasma for bone regeneration. Materials 10:E831. 10.3390/ma10070831 28773189 PMC5551874

[B76] Li Z. Liu Y. S. Ye H. Q. Liu Y. S. Hu W. J. Zhou Y. S. (2017). [Diagnossis and treatment of complicated anterior teeth esthetic defects by combination of whole-process digital esthetic rehabilitation with periodontic surgery]. Beijing Da Xue Xue Bao 49, 71–75. 28203007

[B77] Ligon S. C. Liska R. Stampfl J. Gurr M. Mülhaupt R. (2017). Polymers for 3D printing and customized additive manufacturing. Chem. Rev. 117, 10212–10290. 10.1021/acs.chemrev.7b00074 28756658 PMC5553103

[B78] Liu Y. Song F. Wu S. He S. Meng M. Lv C.. (2017). Protein and mRNA expressions of IL-6 and its key signaling factors under orthodontic forces in mice: an *in-vivo* study. Am. J. Orthod. Dentofacial Orthop. 152, 654–662. 10.1016/j.ajodo.2017.03.026 29103443

[B79] Ma Y. Ji Y. Huang G. Ling K. Zhang X. Xu F. (2015). Bioprinting 3D cell-laden hydrogel microarray for screening human periodontal ligament stem cell response to extracellular matrix. Biofabrication 7:044105. 10.1088/1758-5090/7/4/044105 26696269

[B80] Mangano F. G. De Franco M. Caprioglio A. Macchi A. Piattelli A. Mangano C. (2014). Immediate, non-submerged, root-analogue direct laser metal sintering (DLMS) implants: a 1-year prospective study on 15 patients. Lasers Med. Sci. 29, 1321–1328. 10.1007/s10103-013-1299-0 23494103

[B81] Mardis N. J. (2018). Emerging technology and applications of 3D printing in the medical field. Mo. Med. 115, 368–373. 30228770 PMC6140256

[B82] Marsh J. L. Vannier M. W. (1983). Surface imaging from computerized tomographic scans. Surgery 94, 159–165. 6879436

[B83] Martorelli M. Gerbino S. Giudice M. Ausiello P. (2013). A comparison between customized clear and removable orthodontic appliances manufactured using RP and CNC techniques. Dent. Mater. 29, e1–10. 10.1016/j.dental.2012.10.011 23140842

[B84] Mazzoli A. (2013). Selective laser sintering in biomedical engineering. Med. Biol. Eng. Comput. 51, 245–256. 10.1007/s11517-012-1001-x 23250790

[B85] Miller E. D. Phillippi J. A. Fisher G. W. Campbell P. G. Walker L. M. Weiss L. E. (2009). Inkjet printing of growth factor concentration gradients and combinatorial arrays immobilized on biologically-relevant substrates. Comb. Chem. High Throughput Screen 12, 604–618. 10.2174/138620709788681907 19601758

[B86] Moroni L. Boland T. Burdick J. A. De Maria C. Derby B. Forgacs G.. (2017). Biofabrication: a guide to technology and terminology. Trends Biotechnol. 36, 384–402 10.1016/j.tibtech.2017.10.015 29137814

[B87] Murphy S. V. Atala A. (2014). 3D bioprinting of tissues and organs. Nat. Biotechnol. 32, 773–785. 10.1038/nbt.2958 25093879

[B88] Murray P. E. Garcia-Godoy F. Hargreaves K. M. (2007). Regenerative endodontics: a review of current status and a call for action. J. Endod. 33, 377–390. 10.1016/j.joen.2006.09.013 17368324

[B89] Neunzehn J. Weber M.-T. Wittenburg G. Lauer G. Hannig C. Wiesmann H.-P. (2014). Dentin-like tissue formation and biomineralization by multicellular human pulp cell spheres *in vitro*. Head Face Med. 10:25. 10.1186/1746-160X-10-25 24946771 PMC4074584

[B90] Nogueira S. S. Ogle R. E. Davis E. L. (1999). Comparison of accuracy between compression- and injection-molded complete dentures. J. Prosthet. Dent. 82, 291–300. 10.1016/S0022-3913(99)70083-1 10479255

[B91] Normando D. (2014). 3D orthodontics-from verne to shaw. Dental Press J. Orthod. 19, 12–13. 10.1590/2176-9451.19.6.012-013.edt 25628074 PMC4347405

[B92] Oh J.-H. (2018). Recent advances in the reconstruction of cranio-maxillofacial defects using computer-aided design/computer-aided manufacturing. Maxillofac. Plast. Reconstr. Surg. 40:2. 10.1186/s40902-018-0141-9 29430438 PMC5797724

[B93] Osman R. B. van der Veen A. J. Huiberts D. Wismeijer D. Alharbi N. (2017). 3D-printing zirconia implants; a dream or a reality? An *in-vitro* study evaluating the dimensional accuracy, surface topography and mechanical properties of printed zirconia implant and discs. J. Mech. Behav. Biomed. Mater. 75, 521–528. 10.1016/j.jmbbm.2017.08.018 28846981

[B94] Pacifici A. Laino L. Gargari M. Guzzo F. Velandia Luz A. Polimeni A.. (2018). Decellularized hydrogels in bone tissue engineering: a topical review. Int. J. Med. Sci. 15, 492–497. 10.7150/ijms.22789 29559838 PMC5859772

[B95] Philippe B. (2013). Custom-made prefabricated titanium miniplates in Le Fort I osteotomies: principles, procedure and clinical insights. Int. J. Oral Maxillofac. Surg. 42, 1001–1006. 10.1016/j.ijom.2012.12.013 23602483

[B96] Pilipchuk S. P. Monje A. Jiao Y. Hao J. Kruger L. Flanagan C. L.. (2016). Integration of 3D printed and micropatterned polycaprolactone scaffolds for guidance of oriented collagenous tissue formation *in vivo*. Adv. Healthc. Mater. 5, 676–687. 10.1002/adhm.201500758 26820240 PMC4805502

[B97] Polley J. W. Figueroa A. A. (2013). Orthognathic positioning system: intraoperative system to transfer virtual surgical plan to operating field during orthognathic surgery. J. Oral Maxillofac. Surg. 71, 911–920. 10.1016/j.joms.2012.11.004 23312847

[B98] Rahman S. U. Nagrath M. Ponnusamy S. Arany P. R. (2018). Nanoscale and macroscale scaffolds with controlled-release polymeric systems for dental craniomaxillofacial tissue engineering. Materials 11:E1478. 10.3390/ma11081478 30127246 PMC6120038

[B99] Rasperini G. Pilipchuk S. P. Flanagan C. L. Park C. H. Pagni G. Hollister S. J.. (2015). 3D-printed Bioresorbable Scaffold for Periodontal Repair. J. Dent. Res. 94, 153S−7S. 10.1177/0022034515588303 26124215

[B100] Revilla León M. Klemm I. M. García-Arranz J. Özcan M. (2017). 3D metal printing - additive manufacturing technologies for frameworks of implant-borne fixed dental prosthesis. Eur. J. Prosthodont. Restor. Dent. 25, 143–147. 10.1922/EJPRD_RevillaLeon05 28869368

[B101] Reynolds M. Reynolds M. Adeeb S. El-Bialy T. (2011). 3-d volumetric evaluation of human mandibular growth. Open Biomed. Eng. J. 5, 83–89. 10.2174/1874120701105010083 22046201 PMC3204416

[B102] Rischen R. J. Breuning K. H. Bronkhorst E. M. Kuijpers-Jagtman A. M. (2013). Records needed for orthodontic diagnosis and treatment planning: a systematic review. PLoS ONE 8:e74186. 10.1371/journal.pone.0074186 24265669 PMC3827061

[B103] Rodrigues C. T. Oliveira-Santos C. de Bernardineli N. Duarte M. A. H. Bramante C. M. Minotti-Bonfante P. G.. (2016). Prevalence and morphometric analysis of three-rooted mandibular first molars in a Brazilian subpopulation. J. Appl. Oral Sci. 24, 535–542. 10.1590/1678-775720150511 27812625 PMC5083032

[B104] Saijo H. Igawa K. Kanno Y. Mori Y. Kondo K. Shimizu K.. (2009). Maxillofacial reconstruction using custom-made artificial bones fabricated by inkjet printing technology. J. Artif. Organs 12, 200–205. 10.1007/s10047-009-0462-7 19894095

[B105] Salmi M. Paloheimo K.-S. Tuomi J. Ingman T. Mäkitie A. (2013). A digital process for additive manufacturing of occlusal splints: a clinical pilot study. J. R. Soc. Interface 10:20130203. 10.1098/rsif.2013.0203 23614943 PMC3673156

[B106] Salmi M. Tuomi J. Sirkkanen R. Ingman T. Mäkitie A. (2012). Rapid tooling method for soft customized removable oral appliances. Open Dent. J. 6, 85–89. 10.2174/1874210601206010085 22615719 PMC3355367

[B107] Sándor G. K. Numminen J. Wolff J. Thesleff T. Miettinen A. Tuovinen V. J.. (2014). Adipose stem cells used to reconstruct 13 cases with cranio-maxillofacial hard-tissue defects. Stem Cells Transl. Med. 3, 530–540. 10.5966/sctm.2013-0173 24558162 PMC3973720

[B108] Schweiger J. Beuer F. Stimmelmayr M. Edelhoff D. Magne P. Güth J. F. (2016). Histo-anatomic 3D printing of dental structures. Br. Dent. J. 221, 555–560. 10.1038/sj.bdj.2016.815 27811863

[B109] Seifi M. Kazemi B. Kabiri S. Badiee M. (2017). Analysis of transforming growth factor- β1 expression in resorptive lacunae following orthodontic tooth movement in an animal model. Cell J. 19, 278–282. 10.22074/cellj.2016.4218 28670520 PMC5412786

[B110] Shafiee A. Atala A. (2016). Printing technologies for medical applications. Trends Mol. Med. 22, 254–265. 10.1016/j.molmed.2016.01.003 26856235

[B111] Shah P. Chong B. S. (2018). 3D imaging, 3D printing and 3D virtual planning in endodontics. Clin. Oral Investig. 22, 641–654. 10.1007/s00784-018-2338-9 29330656

[B112] Stanek M. Manas D. Manas M. Navratil J. (2012). Comparison of different rapid prototyping methods. Int. J. Math. Comput. Simul. 6, 550–557.

[B113] Stansbury J. W. Idacavage M. J. (2016). 3D printing with polymers: challenges among expanding options and opportunities. Dent. Mater. 32, 54–64. 10.1016/j.dental.2015.09.018 26494268

[B114] Tack P. Victor J. Gemmel P. Annemans L. (2016). 3D-printing techniques in a medical setting: a systematic literature review. Biomed. Eng. 15:115. 10.1186/s12938-016-0236-4 27769304 PMC5073919

[B115] Tardieu P. B. Vrielinck L. Escolano E. Henne M. Tardieu A. (2007). Computer-assisted implant placement: scan template, simplant, surgiguide, and SAFE system. Int. J. Periodontics Restorative Dent. 27, 141–149. 17514886

[B116] Torabi K. Farjood E. Hamedani S. (2015). Rapid prototyping technologies and their applications in prosthodontics, a review of literature. J. Dent. 16, 1–9. 25759851 PMC4345107

[B117] Torres K. Staśkiewicz G. Sniezynski M. Drop A. Maciejewski R. (2011). Application of rapid prototyping techniques for modelling of anatomical structures in medical training and education. Folia Morphol. 70, 1–4. 21604245

[B118] Tsai K.-Y. Lin H.-Y. Chen Y.-W. Lin C.-Y. Hsu T.-T. Kao C.-T. (2017). Laser sintered magnesium-calcium silicate/poly-ε-caprolactone scaffold for bone tissue engineering. Materials 10:65. 10.3390/ma10010065 28772425 PMC5344575

[B119] Uçar Y. Aysan Meriç I. Ekren O. (2018). Layered manufacturing of dental ceramics: fracture mechanics, microstructure, and elemental composition of lithography-sintered ceramic. J. Prosthodont. 10.1111/jopr.12748 29430836

[B120] Unkovskiy A. Spintzyk S. Brom J. Huettig F. Keutel C. (2018). Direct 3D printing of silicone facial prostheses: a preliminary experience in digital workflow. J Prosthet Dent. 120, 303–308. 10.1016/j.prosdent.2017.11.007 29429837

[B121] van Steenberghe D. Glauser R. Blombäck U. Andersson M. Schutyser F. Pettersson A.. (2005). A computed tomographic scan-derived customized surgical template and fixed prosthesis for flapless surgery and immediate loading of implants in fully edentulous maxillae: a prospective multicenter study. Clin. Implant. Dent. Relat. Res. 7(Suppl 1), S111–S120. 10.1111/j.1708-8208.2005.tb00083.x 16137096

[B122] Visscher D. O. Farré-Guasch E. Helder M. N. Gibbs S. Forouzanfar T. van Zuijlen P. P.. (2016). Advances in bioprinting technologies for craniofacial reconstruction. Trends Biotechnol. 34, 700–710. 10.1016/j.tibtech.2016.04.001 27113634

[B123] Wang G. Li J. Khadka A. Hsu Y. Li W. Hu J. (2012). CAD/CAM and rapid prototyped titanium for reconstruction of ramus defect and condylar fracture caused by mandibular reduction. Oral Surg. Oral Med. Oral Pathol. Oral Radiol. 113, 356–361. 10.1016/j.tripleo.2011.03.034 22676828

[B124] Wang X.-F. Song Y. Liu Y.-S. Sun Y.-C. Wang Y.-G. Wang Y.. (2016). Osteogenic differentiation of three-dimensional bioprinted constructs consisting of human adipose-derived stem cells *in vitro* and *in vivo*. PLoS ONE 11:e0157214. 10.1371/journal.pone.0157214 27332814 PMC4917247

[B125] Wang Y. Zhang Y. Zhang Z. Li X. Pan J. Li J. (2018). Reconstruction of mandibular contour using individualized high-density porous polyethylene (Medpor^®^) implants under the guidance of virtual surgical planning and 3D-printed surgical templates. Aesthetic Plast. Surg. 42, 118–125. 10.1007/s00266-017-1029-2 29260271

[B126] Wenz A. Borchers K. Tovar G. E. M. Kluger P. J. (2017). Bone matrix production in hydroxyapatite-modified hydrogels suitable for bone bioprinting. Biofabrication 9:044103. 10.1088/1758-5090/aa91ec 28990579

[B127] Werz S. M. Zeichner S. J. Berg B. I. Zeilhofer H. F. Thieringer F. (2018). 3D printed surgical simulation models as educational tool by maxillofacial surgeons. Eur. J. Dent. Educ. 22, e500–e505. 10.1111/eje.12332 29479802

[B128] Wilde F. Schramm A. (2016). [Computer-aided reconstruction of the facial skeleton : planning and implementation in clinical routine]. HNO 64, 641–649. 10.1007/s00106-016-0220-0 27525666

[B129] Witjes M. J. H. Schepers R. H. Kraeima J. (2018). Impact of 3D virtual planning on reconstruction of mandibular and maxillary surgical defects in head and neck oncology. Curr. Opin. Otolaryngol. Head Neck Surg. 26, 108–114. 10.1097/MOO.0000000000000437 29470184

[B130] Wurm M. C. Möst T. Bergauer B. Rietzel D. Neukam F. W. von Cifuentes S. C.. (2017). *In-vitro* evaluation of Polylactic acid (PLA) manufactured by fused deposition modeling. J. Biol. Eng. 11:29. 10.1186/s13036-017-0073-4 28919925 PMC5594599

[B131] Xiao L. Tsutsui T. (2013). Characterization of human dental pulp cells-derived spheroids in serum-free medium: stem cells in the core. J. Cell. Biochem. 114, 2624–2636. 10.1002/jcb.24610 23794488

[B132] Xu H. Han D. Dong J.-S. Shen G.-X. Chai G. Yu Z.-Y.. (2010). Rapid prototyped PGA/PLA scaffolds in the reconstruction of mandibular condyle bone defects. Int. J. Med. Robot. 6, 66–72. 10.1002/rcs.290 20013824

[B133] Xu H. H. Wang P. Wang L. Bao C. Chen Q. Weir M. D.. (2017). Calcium phosphate cements for bone engineering and their biological properties. Bone Res. 5:17056. 10.1038/boneres.2017.56 29354304 PMC5764120

[B134] Xu J. Hu M. (2017). [A preliminary study of three-dimensional bio-printing by polycaprolactone and periodontal ligament stem cells]. Zhonghua Kou Qiang Yi Xue Za Zhi 52, 238–242. 10.3760/cma.j.issn.1002-0098.2017.04.009 28412790

[B135] Yang Z.-H. Jin F. Zhang X.-J. Liu X. Zhang Y.-F. Liu J.-Q.. (2010). A novel possible strategy based on self-assembly approach to achieve complete periodontal regeneration. Artif. Organs 34, 603–609. 10.1111/j.1525-1594.2009.00991.x 20545657

[B136] Yao Q. Cosme J. G. L. Xu T. Miszuk J. M. Picciani P. H. S. Fong H.. (2017). Three dimensional electrospun PCL/PLA blend nanofibrous scaffolds with significantly improved stem cells osteogenic differentiation and cranial bone formation. Biomaterials 115, 115–127. 10.1016/j.biomaterials.2016.11.018 27886552 PMC5181114

[B137] Yusa K. Yamanochi H. Takagi A. Iino M. (2017). Three-dimensional printing model as a tool to assist in surgery for large mandibular tumour: a case report. J. Oral Maxillofac. Res. 8:e4. 10.5037/jomr.2017.8204 28791080 PMC5541989

[B138] Yuzbasioglu E. Kurt H. Turunc R. Bilir H. (2014). Comparison of digital and conventional impression techniques: evaluation of patients' perception, treatment comfort, effectiveness and clinical outcomes. BMC Oral Health 14:10. 10.1186/1472-6831-14-10 24479892 PMC3913616

[B139] Zhang Y. S. Yue K. Aleman J. Moghaddam K. M. Bakht S. M. Yang J.. (2017). 3D bioprinting for tissue and organ fabrication. Ann. Biomed. Eng. 45, 148–163. 10.1007/s10439-016-1612-8 27126775 PMC5085899

[B140] Zhou X. Castro N. J. Zhu W. Cui H. Aliabouzar M. Sarkar K.. (2016). Improved human bone marrow mesenchymal stem cell osteogenesis in 3D bioprinted tissue scaffolds with low intensity pulsed ultrasound stimulation. Sci. Rep. 6:32876. 10.1038/srep32876 27597635 PMC5011779

